# Optimal Post-Operative Nalbuphine Dose Regimen: A Randomized Controlled Trial in Patients with Laparoscopic Cholecystectomy

**DOI:** 10.3390/medicina60020195

**Published:** 2024-01-23

**Authors:** Guan-Yu Chen, Kung-Kai Kuo, Shih-Chang Chuang, Kuang-Yi Tseng, Shen-Nien Wang, Wen-Tsan Chang, Kuang-I Cheng

**Affiliations:** 1 Department of Anesthesiology, Kaohsiung Medical University Hospital, Kaohsiung Medical University, Kaohsiung 807, Taiwan; kindtaco@gmail.com (G.-Y.C.); deepbluetseng@gmail.com (K.-Y.T.); 2 Department of Anesthesiology, College of Medicine, Kaohsiung Medical University, Kaohsiung 807, Taiwan; 3 Division of General and Digestive Surgery, Department of Surgery, Kaohsiung Medical University Hospital, Kaohsiung Medical University, Kaohsiung 807, Taiwan; kuoksfo168@gmail.com (K.-K.K.); chuangsc@cc.kmu.edu.tw (S.-C.C.); snwang@kmu.edu.tw (S.-N.W.); wtchang@kmu.edu.tw (W.-T.C.)

**Keywords:** laparoscopic cholecystectomy, patient control analgesia, post-operative pain, nalbuphine

## Abstract

*Background* and *Objectives*: Optimal opioid analgesia is an excellent analgesia that does not present unexpected adverse effects. Nalbuphine, acting on the opioid receptor as a partial mu antagonist and kappa agonist, is considered a suitable option for patients undergoing laparoscopic surgery. Therefore, we aim to investigate the appropriate dosage of nalbuphine for post-operative pain management in patients with laparoscopic cholecystectomy. *Materials and Methods*: Patients were randomly categorized into low, medium, and high nalbuphine groups. In each group, a patient control device for post-operative pain control was programed with a low (0.05 mg/kg), medium (0.10 mg/kg), or high (0.20 mg/kg) nalbuphine dose as a loading dose and each bolus dose with a lockout interval of 7 min and without background infusion. Primary and secondary outcomes included the post-operative pain scale and nalbuphine consumption, and episodes of post-operative opioid-related adverse events and satisfactory scores. *Results*: The low-dosage group presented a higher initial self-reported pain score in comparison to the other two groups for the two hours post-op (*p* = 0.039) but presented lower nalbuphine consumption than the other two groups for four hours post-op (*p* = 0.047). There was no significant difference in the analysis of the satisfactory score and adverse events. *Conclusions*: An appropriate administration of nalbuphine could be 0.1 to 0.2 mg/kg at the initial four hours; this formula could be modified to a lower dosage (0.05 mg/kg) in the post-operative management of laparoscopic cholecystectomy.

## 1. Introduction

Multimodal analgesia and opioid sparing concepts have been the hot topic of post-operative pain management. However, opioid administration is still required to mitigate moderate to severe post-operative pain and is not easily replaced, despite opioids possessing inherent limitations of various adverse effects via the activation of central and peripheral opioid receptors [1]. Nalbuphine is a synthetic agonist–antagonist opioid, demonstrated to attenuate mu-opioid receptor-related adverse events such as pruritis, nausea/emesis, constipation, respiratory depression, undesirable sedation, and the development of tolerance and dependence [2]. With several clinical reports, nalbuphine has been recognized as a safe and highly efficacious opioid analgesic that possesses remarkably low narcotic abuse liability [3]. Due to its agonist–antagonist feature and low abuse potential, nalbuphine was removed from the list of controlled substances under the Controlled Substances Act in the United States [4]. As a result, nalbuphine provides an alternative choice to replace strong opioids for the clinical practice of post-operative pain management [5,6].

Some studies have even revealed that the analgesic potency of nalbuphine is equivalent to morphine on a milligram bias [7], although a dispute remains regarding its efficacy [8,9,10,11,12,13]. In clinical intravenous opioid settings, the analgesic effect of nalbuphine 15 mg is almost equivalent to morphine 10 mg or pethidine 120 mg, although minimal or open surgical procedures presenting different degrees of tissue damage might change these conversion rates.

Here, we emphasize that the principle of opioid administration in modern medical practice is to obtain the maximum analgesic effect without intolerable adverse effects while increasing the tendency of minimal invasive procedures and the role of nalbuphine in settings of multi-model analgesia. Although there are several studies of post-operative pain management with nalbuphine, there is limited information demonstrating the appropriate dosages of nalbuphine in minimally invasive surgery. This study evaluated the efficacy, adverse effects, and satisfaction of three different doses of nalbuphine for post-operative pain after elective laparoscopic cholecystectomy.

## 2. Materials and Methods

### 2.1. Study Design and Participants

This was a parallel-group, investigator-initiated, single-blinded randomized controlled trial with three parallel arms. This study received approval from the Institutional Review Board of Kaohsiung Medical University Chung-Ho Memorial Hospital (approval number: KMUHIRB-F(I)-20190070) on 14 June 2019. The most recent iteration of the study protocol (V1.3) received approval on 7 August 2020. This study was prospectively registered with ClinicalTrials.gov (NCT04135534) on 20 October 2019. The trial was conducted in accordance with Good Clinical Practice guidelines and the Declaration of Helsinki in Kaohsiung Medical University Chung-Ho Memorial Hospital, Taiwan. All participants provided written informed consent before participation.

We enrolled adults aged from 20 to 65 years who were scheduled for elective laparoscopic cholecystectomy procedure. Participants were enrolled in this study by study staff members who assessed the eligibility of each patient. Exclusion criteria included allergy or intolerance to drugs relevant to this study, chronic pain history or cancer under regular pain medications, active liver disease, patients who could not cooperate to the evaluation of the survey (dementia or psychosis), and those using hypnosis regularly within the previous three months.

Before surgery, patients were randomly categorized into groups of low (0.05 mg/kg), medium (0.10 mg/kg), or high (0.20 mg/kg) dosage with 1:1:1 ratio, using a computer-generated stratified block randomization technique by gender. Treatment assignments were concealed from patients, the statistician, and evaluators. The anesthesiologists performed the computerized allocation on the day of the surgery, and they did not participate in the patient assessment process at any point. The evaluators involved in data collection were unaware of the specific dosage assignments, and they were blinded to the treatment groups during the study. Additionally, the patients themselves were not informed of their assigned dosages and remained unaware of these assignments while recording their self-report assessments at predetermined time intervals.

### 2.2. Study Treatment

No premedication was given, including hypnotic drug for insomnia or anxiety. Routine intraoperative monitoring included noninvasive blood pressure, electrocardiogram, pulse oxygen saturation, end-tidal concentrations of carbon dioxide and inhaled anesthetics, and body temperature. Invasive arterial pressure was monitored when necessary. In each case, surgery was elective laparoscopic cholecystectomy procedure.

For patients enrolled in this study, anesthesia was induced with propofol, xylocaine, and rocuronium and then maintained with sevoflurane inhalation supplemented with rocuronium, and no NSAIDs (nonsteroidal anti-inflammatory drugs) nor opioids were administrated during operation. After surgery, nalbuphine was administered according to the selected dosage via intravenous patient-controlled device nalbuphine administration. The patient-controlled analgesia program was configured as follows: Nalbuphine was administered at a concentration of 0.4 mg/mL. The loading dose was determined based on randomized groups, with options for low (0.05 mg/kg), medium (0.10 mg/kg), or high (0.20 mg/kg) dosages. The bolus dosage was set at one-third of the loading dose, and each bolus dose had a lockout interval of 7 min, with no background infusion. Patients underwent a minimum of 30 min of monitoring in the post-anesthesia care unit prior to their transfer back to their respective hospital wards.

Patients experiencing post-operative pain that was not well tolerated initially received treatment with patient-controlled analgesia. If the patient continued to experience discomfort that was intolerable, additional analgesics such as nonsteroidal anti-inflammatory drugs (NSAIDs) and other opioids were administered. In the post-anesthesia care unit, pain management was performed by nurse anesthetists and anesthesiologists and was later assigned in the ward to nurses and surgeons. In the wards, patient-controlled analgesia was conducted by nurse anesthetists who evaluated patients post-operative pain twice daily; supplemental analgesics were prescribed by surgeons if the patient could not tolerate the post-operative pain or the side effects of nalbuphine.

The assessors and patients were unaware of dosage assignment, except for the investigators (the in-charge anesthesiologists). In the wards, the primary care nurses and in-charge surgeons were also blinded to the dosage assignment of the patients in this study. Moreover, the enrolled patients in this study were labeled at the computerized provider order entry to prevent violation of the experiment design. The assessor would evaluate the patient after the patient regained his/her consciousness at the post-anesthesia care unit (time zero hour), and visited the patient at the time intervals of 1 h, 2 h, 4 h, 6 h, 24 h, and 36 h after the surgery. However, the initial post-operative pain (time zero hour) score was evaluated 5 min after intravenous nalbuphine loading dose administration according to each dosage program. If the scheduled evaluation time points were too late, the patients were allowed to self-report the measurement with a detailed explanation within the standardized questionnaires, and the assessor would recheck the records.

### 2.3. Statistical Analysis

The primary outcomes were evaluation of post-operative pain with numeric rating scale and nalbuphine consumption within the first 36 h after the procedure. The secondary outcome of this study was defined as a composite measure of post-operative adverse events related to opioid usage and general satisfaction with a scale of 0 to 10 (best) within the first 36 h after surgery. The components of the adverse events were post-operative nausea and vomiting (each rated on a scale of 0–10, 10 means worst), post-operative hypoxemia, Modified Observer’s Assessment of Alertness/Sedation Scale (MOAA/S scale), and post-operative ileus (absence of flatus or stools). Each component of the outcomes was also analyzed separately. Hypoxia and MOAA/S scale were evaluated at the initial period of post-operative 6 h. It is necessary to record the score of satisfactory scale under patients with adequately clear consciousness, and the formal satisfactory score should be recorded in patients in full recovery with the MOAA/S scale.

Based on the pain score data previously acquired, the value for the low-dose group was 4.5 ± 3.0 (mean ± SD), the medium-dose group was 3.7 ± 2.0, and the high-dose group was 2.3 ± 2.0. Power calculation was determing using ANOVA method with the power of 80% and Type 1 error of 5%; the sample size required was calculated as 33 patients in each group.

The study results were presented with continuous variables as mean ± SD, and with categorical variables as a number (percentage). The primary endpoint of this trial comprised pain scores during the follow-up period. The analysis of variance (ANOVA) technique was used to analyze the independent variables, while categorical variables were compared using chi-squared test. A post hoc analysis was conducted to compare the means of all groups using a Tukey–Kramer test, and data analysis was carried out using either SAS software (version 9.4; SAS Institute, Cary, NC, USA) or R software version 4.1.0 (R Foundation for Statistical Computing, Vienna, Austria). The threshold for statistical significance was set at a two-tailed *p*-value of less than 0.05.

## 3. Results

**Figure 1:**
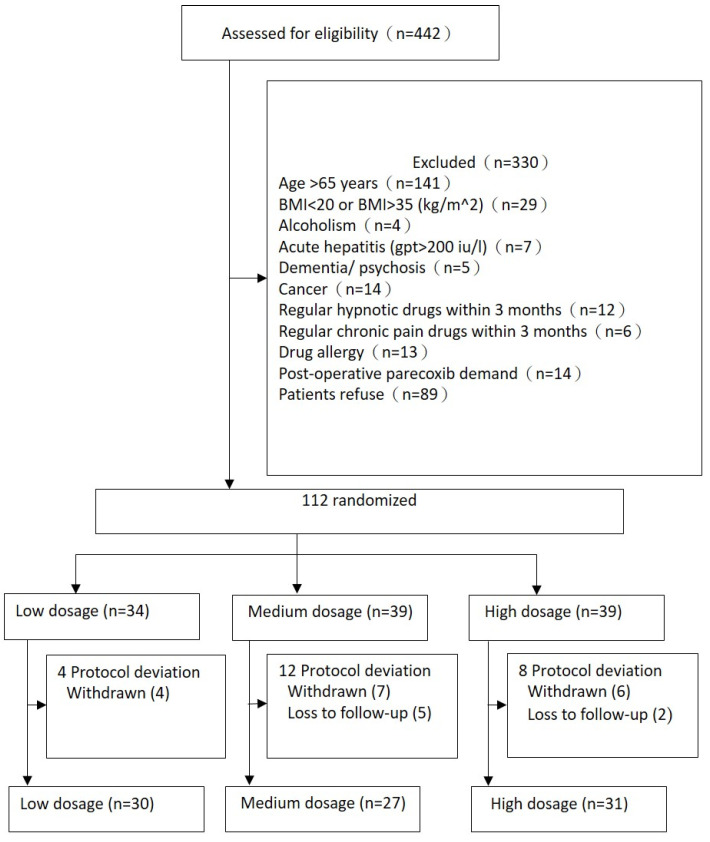
Trial diagram.

Between July 2019 and August 2021, 112 patients arranged for elective laparoscopic cholecystectomy at Kaohsiung Medical University Chung-Ho Memorial Hospital were enrolled in our study and were randomly assigned into low- (*n* = 34), medium- (*n* = 39), or high-dosage (*n* = 39) groups, respectively. There were protocol deviations due to the intolerance to the adverse effects of experiment drugs, due to patients asking for higher medical care quality with multimodal analgesia (there were two participants in the low-dosage group, two participants in the medium-dosage group, and one participant in the high-dosage group who asked for NSAIDs), or patients being lost to follow-up due to early discharge. This study finally enrolled 88 patients. The flow chart of sample enrollment is shown in Figure 1. Follow-up ended in August 2021.

The baseline characteristics of the study patients are listed in Table 1. Baseline characteristics in the three randomized dosages were generally comparable. The demographic data, previous post-operation nausea and vomiting as well as easily induced motion sickness history, incidence of potential risk factors of diabetes, hypertension, cerebrovascular disease, and smoking or drinking habits were comparable among dosages. The American Society of Anesthesiologists’ physical status evaluations of the study population were mostly class II, without a significant difference being observed in these three dosages.

The analysis of pain scores revealed a higher initial self-reported pain score in the low-dosage group compared to the other two dosage groups at the beginning of the post-operative hour following nalbuphine administration and also at the 1 h mark (Table 2; Supplementary Table S1). Moreover, upon considering the low-dosage group, it was observed that they reported an initially lower nalbuphine consumption in comparison to the other two dosage groups (Table 2; Supplementary Table S1). Among the three groups, the pain score revealed a positive correlation with the time interval consumption of nalbuphine in general (Supplementary Table S2). Furthermore, it is noteworthy to mention that our study did not identify significant differences in the time intervals of nalbuphine consumption (Table 2). Nevertheless, it is important to acknowledge that, despite these observations, the initial difference in the primary outcome analysis among the three groups exhibited similar trends of decreasing pain scores after four hours of follow-up. The outcome analysis from the fourth hour and beyond revealed a point of inflection. Regardless of the dosage groups, there was a comparable pain score with a tendency of an even lower total nalbuphine consumption.

Despite the differences in pain scores and nalbuphine consumption among the three groups, there was no significant difference in the satisfactory score and the occurrence of nausea or vomiting among these dosage groups (Table 3). The analysis of flatus passage and defecation, score of MOAA/S scale, and values of end-tidal carbon dioxide analysis presented no significant differences among the low, medium, and high dosages.

## 4. Discussion

This study represents the first investigation to demonstrate the nalbuphine requirements for post-operative pain management in minimally invasive procedures. In this study, the low nalbuphine dosage (0.05 mg/kg) exhibited an initial higher pain score along with lower nalbuphine consumption. However, regardless of the assigned dosage groups, pain scores showed a gradual improvement over time. Additionally, there were no differences in pain scores and nalbuphine consumption at each time interval at or after four hours post-operation. All dosages presented comparable results of the MOAA/S score and satisfactory scores over 36 h. The time of stool passage or flatulence, incidence of vomiting, and itching all showed similar trends. Considering the analysis of primary and secondary outcomes, the results suggest that an optimized administration of nalbuphine could be initiated at 0.1~0.2 mg/kg during the first four hours, and then it should be transitioned to a lower dose (0.05 mg/kg per injection) to meet the decreasing analgesic demands in patients undergoing minimally invasive surgery.

Contrary to the recommendations of several studies that advocate a 0.15~0.3 mg/kg administration of nalbuphine for post-operative pain management [8,9,10,11,12,13], this study did not favor the routine administration of such high dosages of nalbuphine for post-operative pain management in minimally invasive procedures. Firstly, it is important to note that most of those studies were reported before the increasing prevalence of minimally invasive procedures [14]. Consequently, those reports may still adhere to conventional post-operative pain management strategies. However, it has been established that minimally invasive procedures have been shown to reduce the post-operative analgesic dosage and stress response [15,16,17]. This study revealed the consistency of reducing analgesic demands under the pain-reducing minimally invasive surgical techniques [15,16] and reported good efficiency of low-dosage nalbuphine for post-operative pain management. Secondly, despite the initial higher pain score in the low-dosage group, as compared to the other dosages, this dosage matched the non-inferior pain score outcome at about two hours post-operation with lower nalbuphine consumption. In this study, our patients could independently titrate the administration of nalbuphine to control their pain via the patient-controlled analgesia device. This patient-controlled analgesia implies on-demand, intermittent, and self-controlled administration of analgesic agents, providing individual opioid titration with excellent results [18,19,20]. Since this study demonstrated lower total opioid consumption in the low-dosage group and a comparable satisfaction score among the three-dosages groups after two hours post-operation, we infer that low-dosage nalbuphine could potentially be efficient for post-operative pain management in minimally invasive surgery. As a result, we offer a pragmatic approach to evaluate the efficacy of nalbuphine in minimally invasive surgery through pain score assessment and patient-controlled analgesia device, which yield more robust results compared to previous studies.

Post-operative acute pain of laparoscopic cholecystectomy includes components of incisional, non-localized visceral, and referred shoulder pain [21]. A previous study demonstrated visceral pain accounts for the most discomfort as compared to the somatic pain of incisional components [22]. κ-agonists were reported to act as particularly effective analgesics for visceral pain in experimental models, and the properties of nalbuphine are expected to arouse therapeutic interest in various visceral pain conditions, including cholecystectomy [23]. As the patient-controlled analgesia provides a useful device for adjusting analgesic needs [24], our study demonstrated the efficient analgesia effect of nalbuphine for visceral pain with a lower consumption of nalbuphine at two hours post-operation in laparoscopic cholecystectomy, which is also in accordance with the study performed by Liu et al. [25].

This study did not demonstrate that lower nalbuphine usage equals to less side effects. According to Zhao et al.’s reports, once the morphine equivalent dose reaches a threshold, approximately every 4 mg increase in the morphine-equivalent dose is related to increasing the meaningful adverse effects of opioid usage [26]. By using a patient-controlled analgesia device, our study could reach the optimized opioid usage for patients with excellent results [18,19,20]. Moreover, opioid consumption did not reveal an obvious gap of a 4 mg morphine-equivalent dose among dosages, irrespective of the analysis of the accumulated doses or their time intervals (Table 2).

There are limitations in this study that need to be addressed. Firstly, this was a single-center trial, which could attenuate external validity. However, elective laparoscopic cholecystectomy is an almost standardized procedure with similar post-operative care in most countries. Further, compared to multiple-center studies, this single-center study was performed by the same surgical team with the same surgical procedure, and the administration of nalbuphine was standardized in the trial setting. Secondly, the perceived satisfaction (secondary outcome) should consider the adequate dosage of nalbuphine. It is important to assess the psychometric quality of pain outcomes by satisfaction, which cover pain severity, interference with function, affective experience, adverse effects, and perception of care [27]. In the perception of pain care, satisfaction scores showed no significant difference among the low and other dosages, which infers that the initial high dose of nalbuphine changed to low-dose nalbuphine 2 to 4 h post-operation could also be an alternative medical decision for post-operative management in this study. Thirdly, patients who deviated from the protocol were mainly intolerant to the adverse effects and asked for adding NSAIDs into their post-operative pain management. According to the core principle of health care ethics [28], patients could have chosen other analgesic agents for a better experience of post-operative pain management in our study, and there were no significant differences after protocol deviation. Fourthly, as one of the primary outcomes of our study, we did not pre-calculate the sample size concerning nalbuphine consumption. However, we provide the following numerical values: 6.5 ± 5.4 (mean ± standard deviation) for the low-dose group, 10.2 ± 7.3 for the medium-dose group, and 13.1 ± 9.1 for the high-dose group, with a significance level of 0.004. The computed power for these results is 84.4%.

## 5. Conclusions

In conclusion, when solely using nalbuphine for post-operative pain control in patients undergoing laparoscopic cholecystectomy, a high-dose regimen provides better pain control in comparison to the other dosages. However, to optimize a dose regimen for post-operative pain management in laparoscopic cholecystectomy patients, our findings suggest initiating nalbuphine administration at a dosage of 0.2 mg/kg during the initial four hours post-surgery. Subsequently, we advise transitioning to a lower dosage of 0.1–0.05 mg/kg for the continued pain management phase.

## Figures and Tables

**Table 1:** Demographic data between different dosages.

Variables	Low (*n* = 30)	Medium (*n* = 27)	High (*n* = 31)	*p*-Value
Age (yrs)	48.1 ± 10.2	49.9 ± 10.0	44.9 ± 10.6	0.182
Gender (male)	16 (53.3)	17 (63.0)	19 (61.3)	0.726
Body weight (kg)	71.2 ± 10.0	72.9 ± 9.1	70.9 ± 15.1	0.806
Height (cm)	166.7 ± 9.5	165.1 ± 9.1	163.7 ± 7.9	0.416
PONV history	2 (6.7)	0 (0.0)	2 (6.5)	0.395
Motion sickness	4 (13.3)	1 (3.7)	3 (9.68)	0.446
ASA				0.604
I	1 (3.3)	1 (3.7)	2 (6.5)	
II	28 (93.3)	22 (81.5)	26 (83.9)	
III	1 (3.3)	4 (14.8)	3 (9.7)	
HTN	7 (23.3)	6 (22.2)	6 (19.4)	0.927
DM	2 (6.7)	2 (7.4)	3 (9.7)	0.902
CVA	0	0	0	N/A
ESRD	0	0	0	N/A
Smoking	4 (13.3)	1 (3.7)	8 (25.1)	0.059
Drinking	1 (3.3)	1 (3.7)	1 (3.2)	0.995

Data showed as mean ± SD or n (%). N/A = not applicable. ASA = American Society of Anesthesiology classification; CVA = cerebrovascular accident; DM= diabetes mellitus; ESRD = end-stage renal disease; HTN = hypertension; N/A = not applicable; PONV = post-operative nausea and vomiting.

**Table 2:** Primary outcome.

Variables	Low (*n* = 30)	Medium (*n* = 27)	High (*n* = 31)	*p*-Value
Post-op pain score, NRS				
0 h	4.9 ± 3.0	3.7 ± 3.5	2.4 ± 3.1	0.013 *
1 h	5.6 ± 2.4	5.1 ± 2.7	3.8 ± 2.9	0.039 *
2 h	5.0 ± 2.3	4.3 ± 2.3	4.2 ± 2.5	0.314
4 h	4.3 ± 2.3	3.7 ± 2.1	3.7 ± 2.2	0.510
6 h	3.4 ± 2.4	3.3 ± 1.8	3.3 ± 2.1	0.981
24 h	2.8 ± 2.0	2.6 ± 1.7	2.2 ± 1.2	0.402
36 h	2.2 ± 1.8	1.8 ± 1.5	1.7 ± 1.1	0.410
Nalbuphine accumulated consumption (mg)				
1 h	3.4 ± 3.2	7.6 ± 9.8	7.0 ± 7.6	0.064
2 h	6.5 ± 5.4	10.2 ± 7.3	13.1 ± 9.1	0.004 **
4 h	9.4 ± 8.1	13.5 ± 8.3	14.8 ± 9.3	0.047 *
6 h	11.4 ± 10.2	16.2 ± 10.3	18.2 ± 12.6	0.051
24 h	19.5 ± 22.4	26.2 ± 18.5	31.2 ± 28.6	0.161
36 h	23.5 ± 27.9	30.6 ± 23.2	37.4 ± 34.7	0.185
Nalbuphine time interval consumption (mg)				
1~2 h	3.1 ± 2.9	4.1 ± 3.6	6.1 ± 6.1	0.035 *
2~4 h	2.9 ± 3.5	3.3 ± 3.0	1.6 ± 2.8	0.095
4~6 h	1.9 ± 3.0	2.7 ± 3.8	3.5 ± 4.5	0.310
6~24 h	8.1 ± 13.0	10.1 ± 12.8	13.0 ± 20.5	0.495
24~36 h	4.0 ± 7.2	4.3 ± 6.1	6.2 ± 12.3	0.595

Data showed as mean ± SD. * *p* < 0.05. ** *p* < 0.01. NRS= numerical rating scale.

**Table 3:** Secondary outcome.

Variables	Low (*n* = 30)	Medium (*n* = 27)	High (*n* = 31)	*p*-Value
Satisfactory (0–10)				
4 h	7.3 ± 2.2	7.3 ± 2.6	7.4 ± 2.5	0.971
6 h	7.5 ± 2.4	7.6 ± 2.7	7.7 ± 2.5	0.930
24 h	7.8 ± 2.4	7.7 ± 2.9	8.4 ± 2.0	0.514
36 h	7.9 ± 2.4	7.8 ± 3.0	8.5 ± 2.0	0.571
MOAA/SS (0–5)				
0 h	4.8 ± 0.4	4.6 ± 0.5	4.5 ± 0.7	0.221
1 h	5.0 ± 0.2	5.0 ± 0.2	4.9 ± 0.2	0.822
2 h	5.0 ± 0.0	5.0 ± 0.0	5.0 ± 0.0	N/A
4 h	5.0 ± 0.0	5.0 ± 0.0	5.0 ± 0.0	N/A
6 h	5.0 ± 0.0	5.0 ± 0.0	5.0 ± 0.0	N/A
CO_2_ (mmHg)				
0 h	37.2 ± 5.5	37.7 ± 6.7	38.1 ± 5.7	0.848
1 h	36.1 ± 3.8	37.4 ± 5.5	38.2 ± 5.5	0.273
2 h	35.8 ± 5.0	37.5 ± 3.7	37.9 ± 5.8	0.207
4 h	36.4 ± 3.7	38.1 ± 5.5	37.8 ± 5.7	0.384
6 h	37.3 ± 4.4	38.5 ± 5.1	37.1 ± 4.1	0.436
Nausea, *n* (%)	3 (10.0)	3 (11.1)	5 (16.1)	0.743
Vomiting, *n* (%)	2 (6.7)	0 (0.0)	2 (6.5)	0.395
Itchiness, *n* (%)	0 (0.0)	1 (3.7)	0 ( 0.0)	0.319
Stool (hours)	31.2 ± 9.9	36.1 ± 10.5	31.9 ± 7.4	0.107
Flatus (hours)	18.5 ± 7.9	22.7 ± 11.2	21.0 ± 10.5	0.273
Other pain drugs usage, *n* (%)	5 (16.7)	4 (14.8)	5 (16.1)	0.981
Antiemetic drugs, *n* (%)	5 (16.7)	4 (14.8)	4 (12.9)	0.918
Antihistamine drugs, *n* (%)	0 (0.0)	0 (0.0)	0 (0.0)	N/A

Data showed as mean ± SD or *n* (%). N/A = not applicable. MOAA/SS = Modified Observer’s Assessment of Awareness/Sedation Scale.

## Data Availability

The data presented in this study can be made available upon request by contacting the corresponding author.

## Supplementary Materials

The following supporting information can be downloaded at: https://www.mdpi.com/article/10.3390/medicina60020195/s1, Table S1: Post hoc comparisons of all means were performed using a Tukey–Kramer test; Table S2: Correlations of post-operation pain score to nalbuphine time interval consumption between dosages.
