# Supplementary material for: Optimal Post-Operative Nalbuphine Dose Regimen: A Randomized Controlled Trial in Patients with Laparoscopic Cholecystectomy
Source: Medicina (Kaunas). 2024 Jan 23;60(2):195. doi: 10.3390/medicina60020195 (PMC10890534; doi:10.3390/medicina60020195)
Supplement: Supplementary file 1 [file medicina-60-00195-s001.zip › Supplementary table 1 .pdf]

Supplementary table 1. Post hoc comparisons of all means were performed using a Tukey–Kramer test

Variables	Comparisons	P-value
Post-OP pain score		
0 hour	Low to High	0.009
1 hour	Low to High	0.037
2 hours	N/A	N/A
Nalbuphine consumption		
1 hour	N/A	N/A
2 hours	Low to High	0.002
4 hours	Low to High	0.045
1~2 hours	Low to High	0.029
Stool	N/A	N/A
Flatus	N/A	N/A

No significance: P-value > 0.05.   N/A=not applicable.
