# Supplementary material for: Optimal Post-Operative Nalbuphine Dose Regimen: A Randomized Controlled Trial in Patients with Laparoscopic Cholecystectomy
Source: Medicina (Kaunas). 2024 Jan 23;60(2):195. doi: 10.3390/medicina60020195 (PMC10890534; doi:10.3390/medicina60020195)
Supplement: Supplementary file 1 [file medicina-60-00195-s001.zip › Supplementary table 2 .pdf]

Supplementary table 2. Correlations of post-operation pain score to nalbuphine time interval consumption between dosages

		Low (n=30)		Medium (n=27)		High (n=31)	
Post-op pain score	Nalbuphine TIC	Correlation	P-value	Correlation	P-value	Correlation	P-value
1 hours	0~1 hours	0.453	0.012*	0.561	0.002**	0.151	0.416
2 hours	1~2 hours	0.166	0.380	-0.034	0.865	0.352	0.053
4 hours	2~4 hours	0.381	0.038*	0.199	0.321	0.292	0.111
6 hours	4~6 hours	0.361	0.05	0.152	0.450	0.545	0.002**
24 hours	6~24 hours	0.388	0.036*	0.444	0.020*	0.498	0.004**
36 hours	24~36 hours	0.569	0.001**	0.324	0.100	0.026	0.891

TIC= time interval consumption

*p<0.05. **p<0.0
